# The Integrative Imperatives of Locust Phase Polyphenism Research: A Bibliometric Analysis

**DOI:** 10.1093/iob/obag018

**Published:** 2026-05-06

**Authors:** D F Serdo, Z Németh

**Affiliations:** Department of Evolutionary Zoology and Human Biology, University of Debrecen, P. O. Box 400, Debrecen, Hungary; Department of Biology, Ambo University, P. O. Box 19, Ambo, Ethiopia; Juhász-Nagy Pál Doctoral School of Biology and Environmental Sciences, Debrecen University, P. O. Box 400, Debrecen, Hungary; Department of Evolutionary Zoology and Human Biology, University of Debrecen, P. O. Box 400, Debrecen, Hungary

## Abstract

Locust phase polyphenism, a classic example of phenotypic plasticity, has been studied for over a century, generating an extensive body of literature. However, a quantitative synthesis of the field’s structure, evolution, and disparities has been lacking. Here, we present the first comprehensive bibliometric analysis of locust phase polyphenism research, quantitatively mapping its intellectual structure, collaborative networks, and thematic evolution. Analyzing 400 peer-reviewed primary studies published from 1921 through February 2025, we reveal a field at a critical inflection point. Publication trends demonstrate robust growth since the 1990s, reflecting the refinement of classical methods and the emergence of molecular and neurobiological approaches. However, this expansion rests upon a narrow foundation: research concentrates almost exclusively on two model species—*Schistocerca gregaria* and *Locusta migratoria*—while ecologically important non-model taxa remain critically understudied. Co-authorship network analysis exposes significant geographical disparities: research leadership concentrates in a small number of countries (UK, China, Japan, Belgium, Israel, Kenya, Germany, and USA), while most other locust-affected regions across the Sahel, the Horn of Africa, and the Middle East remain peripherally integrated. Keyword co-occurrence mapping identifies critical thematic blind spots within the phase polyphenism literature, including a complete absence of explicit climate change framing and limited representation of socioeconomic dimensions and non-model species. The field thus exhibits a notable disconnect: deep mechanistic knowledge of how phase change occurs exists alongside limited understanding of when and why outbreaks occur under changing environmental conditions. Transformative advances require strategic reorientation: from model-organism focus to comparative ecology, from episodic collaboration to equitable partnership with range-state scientists, from descriptive mechanism to functional validation, and from neglected frontiers (microbiome, epigenetics, and climate) to systematic investigation. This bibliometric mapping provides an evidence-based framework to guide future research toward greater impact on global food security.

## Resumen

El polifenismo de fases en langostas, un ejemplo clásico de plasticidad fenotípica, ha sido estudiado durante más de un siglo, lo que ha generado una extensa literatura científica. Sin embargo, aún se carece de una síntesis cuantitativa sobre la estructura, evolución y disparidades de estecampo. Aquí presentamos el primer análisis bibliométrico exhaustivo sobre la investigación del polifenismo de fases en langostas, mapeando cuantitativamente su estructura intelectual, redes de colaboración y evolución temática. Analizando 400 estudios primarios revisados por pares publicados desde 1921 hasta febrero de 2025, revelamos el campo en un punto de inflexión crítico. Las tendencias de publicación demuestran un crecimiento robusto desde la década de 1990, reflejando el refinamiento de métodos clásicos y el surgimiento de estudios con enfoques moleculares y neurobiológicos. Sin embargo, esta expansión se asienta sobre un fundamento estrecho: la investigación se concentra casi exclusivamente en dos especies modelo—*Schistocerca gregaria* y *Locusta migratoria*—mientras que otros taxones no-modelo ecológicamente importantes permanecen críticamente subestudiados. El análisis de redes de coautoría expone disparidades geográficas significativas: el liderazgo en investigación se concentra en un pequeño número de países (Reino Unido, China, Japón, Bélgica, Israel, Kenia, Alemania, EE. UU.), mientras que la mayoría de otras regiones afectadas por langostas como lo son el Sahel, el Cuerno de África y el Medio Oriente permanecen integradas periféricamente. El mapeo de co-ocurrencia de palabras clave identifica diversos vacíos temáticos críticos en la literatura sobre el polifenismo de fases, incluyendo la ausencia total de un marco explícito sobre cambio climático y una representación limitada de las dimensiones socioeconómicas y de especies no-modelo. Éste campo presenta, por tanto, una desconexión notable: existe un conocimiento mecanístico profundo sobrecómo ocurre el cambio de fase, pero sigue siendo limitado el entendimiento de cuándo y por qué se producenlos brotes bajo condiciones ambientales cambiantes. Avances transformativos requieren de una reorientación estratégica: desde el enfoque centrado en organismos modelo hacia la ecología comparativa; desde la colaboración episódica hacia una asociación equitativa con científicos de los países del área de distribución de las plagas; desde mecanismos descriptivos hacia la validación funcional; y desde fronteras poco exploradas (microbioma, epigenética, clima) hacia una investigación sistemática. Este mapeo bibliométrico proporciona entonces, un marco basado en evidencia para guiar investigaciones futuras hacia un mayor impacto en la seguridad alimentaria global.

## Introduction

Locusts are species of short-horned grasshoppers (Orthoptera: Acrididae) exhibiting a pronounced, density-dependent phenotypic plasticity known as phase polyphenism. This adaptive mechanism enables the formation of large migratory swarms capable of causing substantial economic damage and posing significant challenges to food security across Africa, the Middle East, Asia, and Australia (Simpson et al. 1999; Simpson and Sword 2008; Pener and Simpson 2009; Cullen et al. 2017). The core feature of this polyphenism is the transition from a solitarious, cryptic individual into a gregarious one attracted to conspecifics, resulting in cohesive nymphal bands and migratory adult swarms. This transformation involves coordinated changes across behavior (Roessingh et al. 1993; Buhl et al. 2006; Anstey et al. 2009; Gray et al. 2009; Rogers et al. 2014), morphology (Dirsh 1951; Albrecht and Blackith 1957; Symmons 1969; Maeno et al. 2004), physiology (Ayali and Pener 1992; Fuchs et al. 2003), neurobiology (Matheson et al. 2004; Ott and Rogers 2010), endocrinology (Pener and Lazarovici 1979; Dale and Tobe 1988; Tanaka 2000a; Tawfik et al. 2000), molecular biology (Kang et al. 2004; Chen et al. 2010; Badisco, Ott et al. 2011; Bakkali et al. 2024), and ecology (Collett et al. 1998; Despland et al. 2000, 2004; Babah and Sword 2004). This change is triggered primarily by population density (Pener and Simpson 2009), with other environmental conditions—including resource distribution, humidity, temperature, and substrate color (Ellis 1964; Collett et al. 1998; Despland and Simpson 2000a; Tanaka 2003; Babah and Sword 2004; Maeno and Tanaka 2007; Tanaka et al. 2012)—acting as modulating factors that influence the expression and threshold of density-dependent responses.

Since Boris Uvarov’s foundational phase theory in 1921 (Uvarov 1921), extensive research has elucidated these complex mechanisms resulting in over a thousand publications. This extensive research has been synthesized in numerous authoritative reviews (Kennedy 1956; Pener 1991; Simpson et al. 2005; Tanaka 2006; Pener and Simpson 2009; Simpson and Sword 2009; Sword et al. 2010; Song 2011; Wang and Kang 2014; Ernst et al. 2015; Cullen et al. 2017; Ayali 2019; Pflüger and Bräunig 2021; Guo and Kang 2025). However, while most previous work has focused on presenting specific aspects or ideas by way of reviewing the relevant literature, the current study is a systematic and quantitative analysis of the research landscape. It examines the field’s historical development, collaborative networks, thematic priorities, and, crucially, its blind spots—a comprehensive approach notably lacking in existing studies. By employing bibliometric analysis, we identify key research trends, emerging topics, and the impact of seminal works. These insights are crucial for recognizing biases, highlighting neglected areas, and guiding future research to address scientific and societal challenges.

While the frequency and scale of locust outbreaks have generally decreased due to improved monitoring and control methods, recent decades have witnessed severe outbreaks—most notably the 2019–2021 East African crisis—raising concerns that climate change and land-use transformations may be reversing these gains and intensifying outbreak dynamics. Addressing these threats effectively depends not only on scientific discoveries but also on the structure of the scientific enterprise that produces them. Key questions include: Is research aligned with pressing needs? Are studies conducted in relevant locations? Is knowledge co-produced with affected communities? To address these issues, this study employs bibliometric analysis of the locust phase polyphenism literature to (1) map the historical trends and evolution of the field; (2) analyze global research networks and collaboration patterns, identifying geographical disparities; (3) identify dominant and emerging thematic focuses through keyword analysis to pinpoint critical knowledge gaps; and (4) provide evidence-based recommendations to reorient research priorities. The findings aim to inform policymakers, research funders, and scientific project leaders seeking to foster a more strategic, equitable, and transdisciplinary research environment.

## Methodology

### Literature search strategies

A systematic literature search was conducted in accordance with the PRISMA 2020 guidelines (Page et al. 2021). Searches were performed across three electronic databases, including PubMed, Web of Science Core Collection, and Scopus. The search encompassed all publications indexed in the selected databases prior to February 2025, with no start date restriction. The search strategy utilized comprehensive nested Boolean operators, combining three core term categories following a systematic approach to search strategy development (Bramer et al. 2018). These terms included: population terms (“locust” OR “Schistocerca” OR “*Locusta migratoria*” OR “Nomadacris” OR “Chortoicetes” OR “Austracris”), phenomenon terms (“phase polyphenism” OR “phase polymorphism” OR “phase change” OR “phase transition” OR “phenotypic plasticity” OR “density-dependent” OR “gregari” OR “solitar,” “phase characteristic”), and trait/outcome terms (“behavi” OR “morphometr” OR, “colo” OR “pheromone” OR “juvenile hormone” OR “corazonin” OR “serotonin” OR “transcriptom” OR “microbiome”). Search strings were adapted for each database’s specific syntax and controlled vocabularies. The exact search terms applied to each database are provided in Supplementary File 1. Search results were limited to studies published in English due to resource constraints. To ensure comprehensive coverage, particularly of older seminal studies that may not be easily captured through database searches, we supplemented database searches with backward and forward citation tracking of key seminal papers identified during the initial search.

### Eligibility criteria

Studies were assessed against predefined eligibility criteria. Eligible studies included all locust species documented to exhibit density-dependent phase polyphenism, following the species lists provided in Song (2011) and Cullen et al. (2017), which distinguish true locusts from swarming grasshoppers without a confirmed phase change. Eligible interventions or exposures encompassed any condition that alters or defines phase state, including but not limited to crowding, sensory stimulation, hormonal application, genetic or epigenetic manipulations, or dietary change. A required comparator was a control or contrasting group essential for defining phase differences, such as gregarious (crowded) versus solitarious (isolated). Eligible outcomes included any measurable phase-related trait across behavioral, morphological, physiological, molecular, epigenetic, microbial, life-history, and theoretical categories. Eligible study designs were primary research articles, including laboratory experiments, field experiments, and observational cohort studies. Theoretical models and simulations were included only if they tested hypotheses against primary empirical data. Narrative reviews, editorials, opinion pieces, and studies lacking primary data were excluded. Furthermore, primary studies on locusts that did not investigate phase-related traits or incorporate a density-dependent component as a central variable were excluded.

### Screening and data extraction process

Duplicate records were removed using Mendeley Desktop (version 1.19.8). Following duplicate removal, two reviewers independently screened the titles and abstracts of all remaining unique records against predefined inclusion and exclusion criteria. At this stage, records were excluded if they clearly did not investigate locust phase polyphenism or lacked a density-dependent component. Full texts of all potentially eligible records were then retrieved. Two reviewers independently assessed each full text against the full eligibility criteria, documenting reasons for exclusion. At this stage, records were excluded if they lacked a phase polyphenism focus, did not include an appropriate comparator, or were confirmed not to be primary research after full-text review. To ensure comprehensive coverage, particularly of older seminal studies, backward and forward citation tracking of key papers identified during full-text assessment was conducted; additional studies identified through this cross-referencing underwent the same two-stage screening process. From the final set of included studies, two reviewers independently extracted metadata using a standardized, pilot-tested form, including bibliometric details (authors, publication year, affiliated countries, and journal), study characteristics (locust species, life stage, and primary research focus), methodological details (experimental design, sample size, and treatment groups), key findings, and thematic categorization. The extracted data were exported in RIS and Excel formats for subsequent bibliometric analysis.

### Data analysis

The bibliometric analysis was conducted using R (v4.5.0) for data processing, statistical computation, and visualization of publication trends, alongside VOSviewer (v1.6.20) for constructing and visualizing bibliometric networks. Prior to analysis, metadata from the final corpus underwent systematic standardization. This involved disambiguating author names, standardizing journal names to their full official titles, and applying a comprehensive thesaurus to author keywords. For studies where author keywords were not provided, relevant terms were extracted from titles and abstracts and used as proxy keywords. The keyword thesaurus merged singular and plural forms, standardized different writing formats (e.g., unifying “density dependent” and “density-dependent”), harmonized closely related terms (e.g., grouping “density dependent phase polyphenism” under “phase polyphenism”), and standardized spelling to American English (e.g., colour to color, behaviour to behavior). The standardized dataset was then subjected to (1) productivity analysis to examine publication trends over time, (2) co-authorship analysis to map collaboration networks between countries and individual researchers, and (3) keyword co-occurrence analysis to identify the frequency and relationships among terms, thereby mapping the field’s intellectual structure. Further analyses identified leading countries by first-author and total authorship counts, explored thematic distributions across countries, and highlighted core journals.

## Results

### Literature search results and study characteristics

**Fig. 1:**
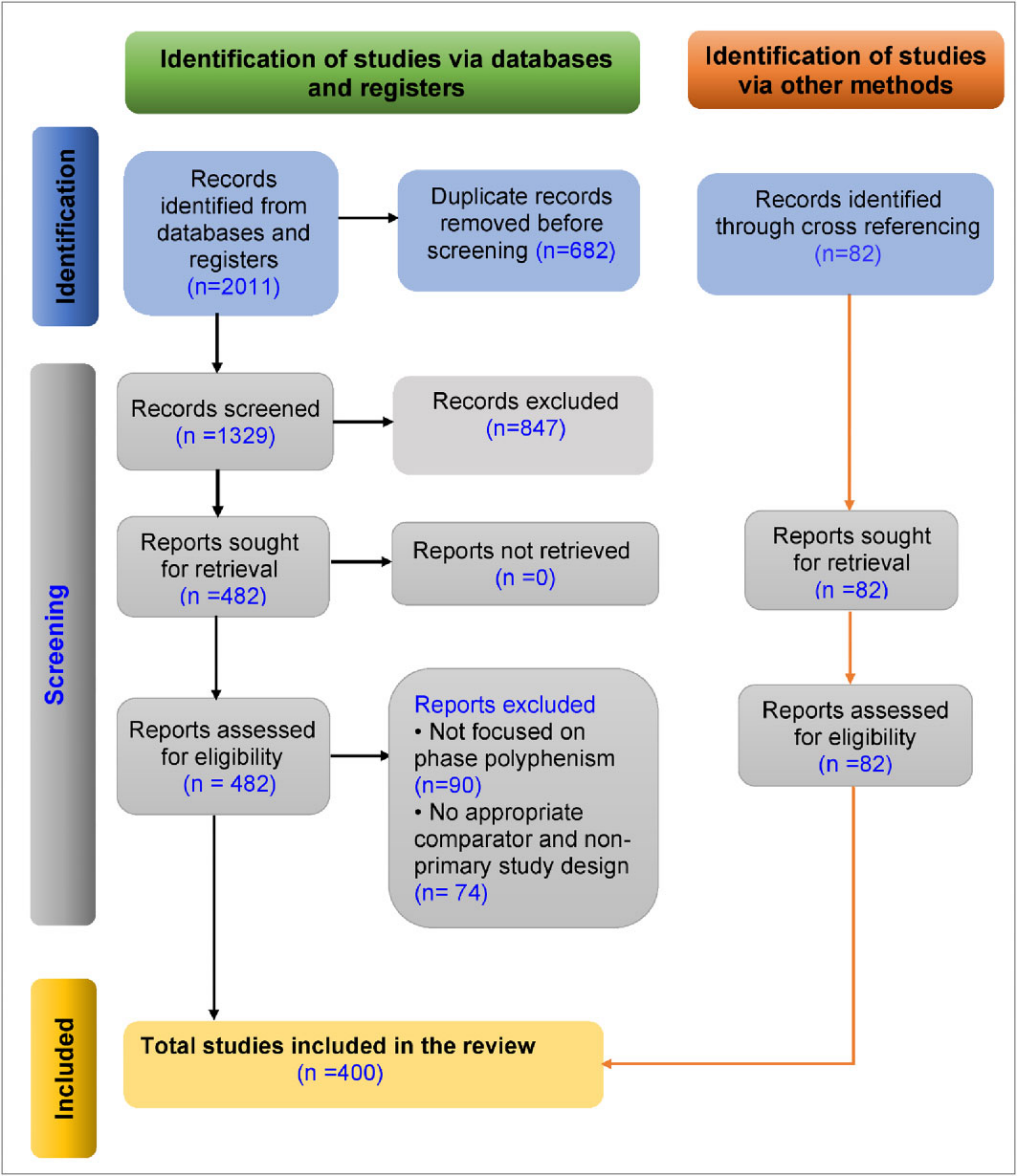
PRISMA flow diagram illustrating the systematic literature search and screening process. The diagram documents the four-stage process (identification, screening, eligibility, and inclusion) from the initial database search, and cross-referencing to the final studies included in the bibliometric dataset.

Our systematic search initially identified 2011 documents. After the removal of 682 duplicates, 1329 records were screened at the title and abstract level, resulting in the exclusion of 847 records that did not meet initial eligibility criteria. Full texts of the remaining 482 potentially eligible records were retrieved and assessed, leading to the exclusion of 164 records for reasons including lack of phase polyphenism focus, absence of an appropriate comparator, or non-primary study design. Cross-referencing of seminal papers identified an additional 82 studies, which underwent the same two-stage screening process; all 82 met the eligibility criteria (Fig. 1). The final bibliometric dataset therefore consisted of 400 unique, peer-reviewed primary studies on locust phase polyphenism, published from 1921 through February 2025. The inclusion of 2025 publications reflects those indexed up to February 2025; therefore, the count for 2025 is partial and should be interpreted as a lower bound of that year’s output. This corpus forms the basis for all subsequent analyses.

### Publication trends over time (1921–2025)

**Fig. 2:**
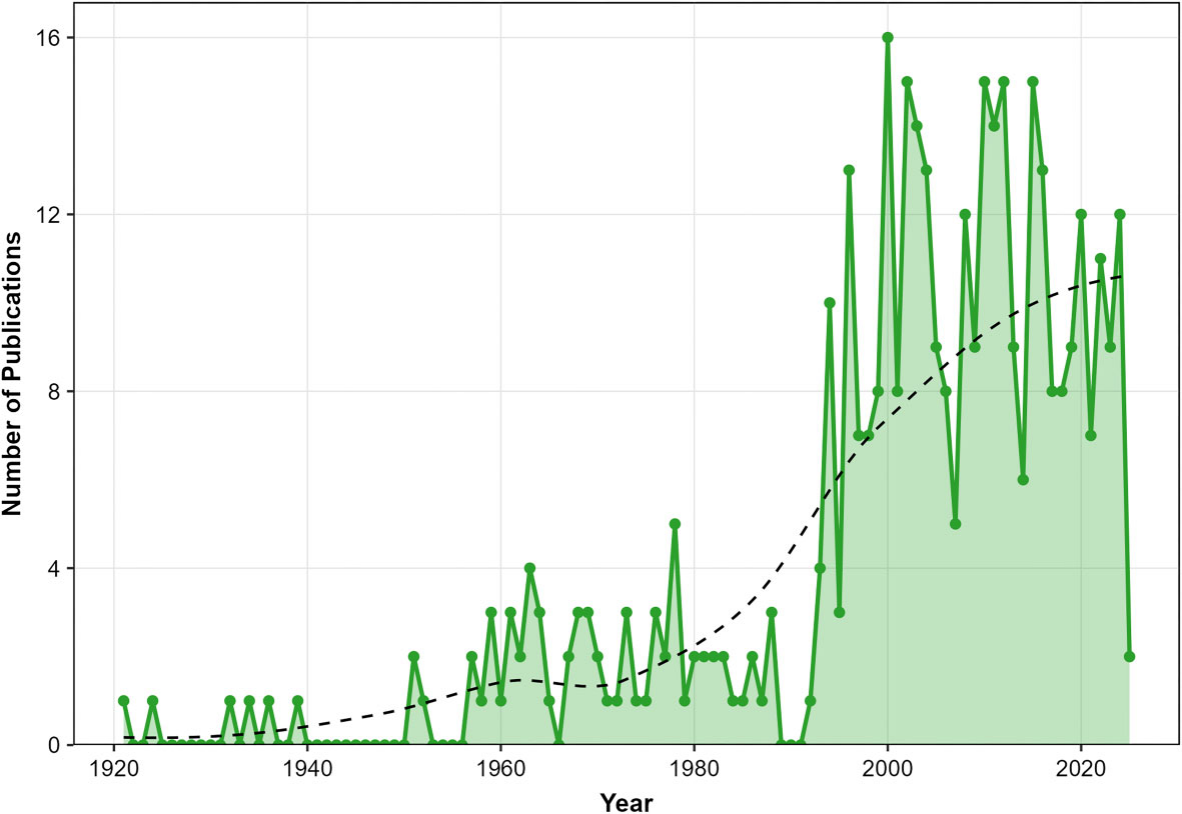
Trends in annual publications on locust phase polyphenism research from 1921 through February 2025.

The publication trajectory of locust phase polyphenism research reveals three distinct periods (see Fig. 2), each characterized by a different growth pattern reflecting shifts in research paradigms, methodological capabilities, institutional infrastructure, and global coordination. From 1921 to 1950, annual publications remained low and flat, reflecting a field in its infancy following the formulation of phase theory: the concept required validation, no dedicated research institutions yet existed, and mechanistic tools were lacking. From 1950 to 1990, publication output increased at a moderate rate. This shift coincided with the institutionalization of locust research through dedicated centers such as the Anti-Locust Research Centre (ALRC) in London and with expanded global coordination by the Food and Agriculture Organization (FAO). It also reflected the application of classical approaches, including behavioral observations and endocrinology. However, growth remained gradual due to the limited throughput and resolution of these methods. From 1990 to 2025, publication output increased dramatically. This acceleration reflects two key developments: the refinement of classical methods into quantitative, high-throughput tools and the emergence of molecular technologies (genomics, transcriptomics, and neurochemistry) alongside interdisciplinary approaches linking individual behavior to collective phenomena. Together, these trends show a clear transition from descriptive validation to mechanistic and integrative, multiscale understanding enabled by modern technologies.

### Global research landscape and collaborative networks

#### Productivity and leadership by country

**Fig. 3:**
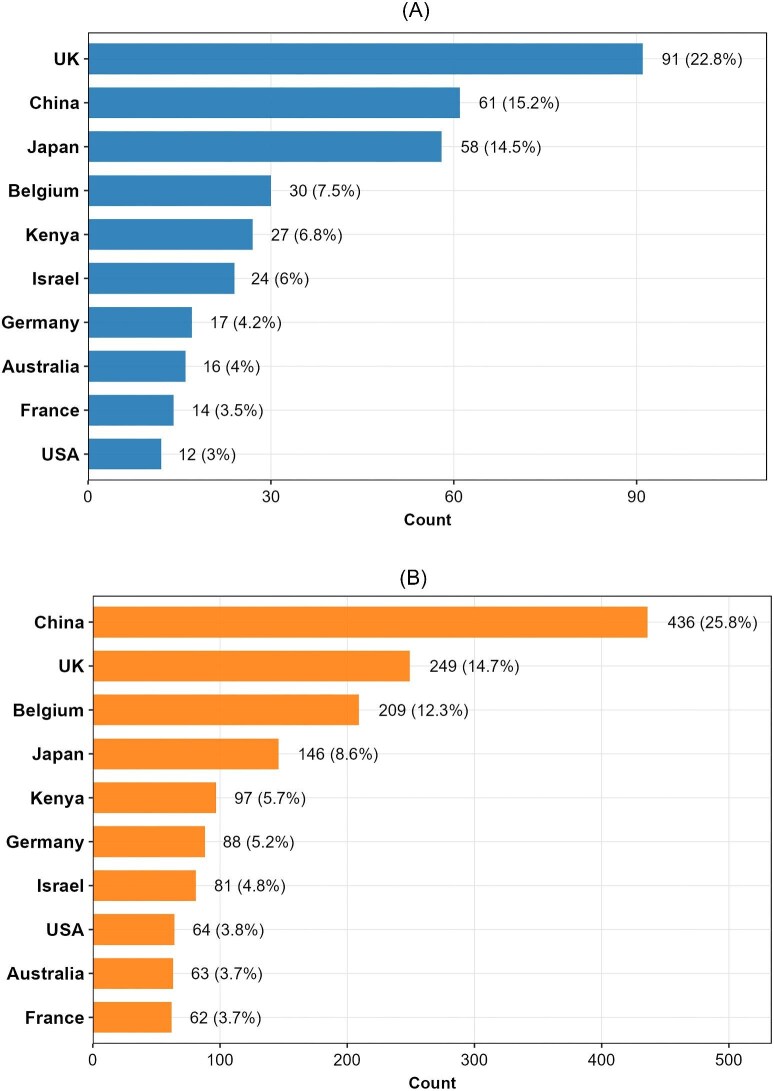
Country-level authorship distribution based on primary affiliation. (**A**) Top ten countries by count of first authors: This figure ranks countries based on the total number of unique first authors whose primary affiliation is in that country. Each researcher is counted once, based on their primary institutional affiliation at the time of publication. (**B**) Top ten countries by count of all authors: This figure ranks countries based on the total number of unique authors whose primary affiliation is in that country, regardless of author position (first, co-author, or last). This metric reflects the overall participation volume.

Analysis of author affiliations reveals a globally distributed but highly stratified research landscape. Figure 3 presents two complementary metrics: first-author contributions and total authorship instances. First-author contributions are used as an indicator of primary contribution to individual studies, while recognizing that authorship conventions vary across disciplines and collaborative contexts. Total authorship instances reflect overall participation and collaboration volume. The relationship between these two metrics provides insight into national research organization, distinguishing leadership-oriented systems from participation-dominated or highly collaborative structures.

**United Kingdom (UK):** The UK ranks first in first-author contributions (91 authors, 22.8%) and second in total authorship instances (249, 14.7%). This positive rank differential (+1) indicates a leadership-skewed authorship structure, where UK-based researchers disproportionately drive study design rather than participating primarily as collaborators. Historically, the UK functioned as a foundational intellectual hub for locust phase theory. Early work established the conceptual framework of phase polyphenism (Uvarov 1921; Uvarov and Hamilton 1936), followed by systematic characterization of behavioral, physiological, and morphometric phase traits. Key advances included the formulation of phase indicators and experimental demonstration of density-dependent and parental effects on phase expression. Field-based ecological studies proposed a three-stage gregarization process—concentration, aggregation, and behavioral gregarization—driven by vegetation structure and microclimatic conditions (Kennedy 1939). Quantitative morphometrics were formalized through the F/C (femur length/head width) ratio as a reliable phase character (Dirsh 1951), while multigenerational experiments demonstrated that parental crowding influences offspring phenotype but can be overridden by nymphal rearing density (Hunter-Jones 1958).

From the mid-20th century onward, UK research diversified into interconnected domains, including behavior, physiology, and social organization. Environmental modulation of phase traits, particularly temperature and humidity effects on morphology and coloration, was systematically characterized (Dudley 1964). A sustained experimental program established the behavioral basis of phase change (Ellis 1959a, 1959b, 1963a, 1963b, 1964; Ellis and Carlisle 1961; Ellis and Pearce 1962), while parallel studies clarified reproductive physiology and endocrine regulation (Norris 1962, 1964; Norris and Pener 1965). Social and population-level processes were further examined through long-term investigations of aggregation and group dynamics (Gillett 1972, 1973a, 1973b, 1975, 1983, 1988; Gillett and Gonta 1978), and population dynamics modeling (Cheke 1978; Holt and Cheke 1996). Research on transgenerational effects demonstrated that parental phase, crowding during mating and oviposition, and nymphal rearing density all exert cumulative gregarizing effects on offspring behavior and coloration (Islam et al. 1994a, 1994b; Bouaïchi et al. 1995). Subsequent work identified a gregarizing factor in egg pod foam and localized it to the female accessory glands (McCaffery et al. 1998; Hägele et al. 2000), and gut bacteria were shown to produce the phenolic components of the cohesion pheromone (Dillon et al. 2002). Maternal phase effects were further linked to endocrine regulation, with gregarious females depositing 5- to 10-fold higher ecdysteroid levels in eggs than solitary females (Hägele et al. 2004).

The UK research has also developed an integrative neuroethological framework linking sensory input to behavioral phase transition. Foundational work established a quantitative behavioral phase index based on multiple parameters, including locomotion, grooming, and attraction to conspecifics (Roessingh et al. 1993), enabling precise measurement of phase state and its temporal dynamics (Roessingh and Simpson 1994). Systematic dissection of sensory modalities demonstrated that mechanical stimulation is a powerful gregarizing stimulus, with visual and olfactory cues also contributing (Hägele and Simpson 2000). Experimental work further demonstrated that tactile stimulation of the hind femur acts as a primary trigger of gregarization (Simpson et al. 2001; Rogers et al. 2003), with serotonin identified as a key mediator of rapid behavioral switching (Rogers et al. 2004; Anstey et al. 2009). In parallel, the UK research has advanced the understanding of neural plasticity and proprioceptive processing, providing mechanistic insight into how sensory information is integrated at the neural level (Matheson et al. 2004; Rogers et al. 2007, 2010; Blackburn et al. 2010; Ott and Rogers 2010; Ott et al. 2012; Mallon et al. 2016). Biomechanical analyses further revealed that solitarious locusts possess enhanced jumping performance due to larger femoral muscle volume and more compliant energy-storage structures (Rogers et al. 2016).

At the collective level, the UK-led studies have established fundamental principles of swarm behavior. These include the identification of a density-dependent transition from disordered movement to coordinated marching (Buhl et al. 2006), the role of nutritional state in modulating social interaction and swarm formation (Bazazi et al. 2011), and theoretical models explaining density-dependent aggregation through predator–prey interactions (Reynolds et al. 2009). Complementary research has linked behavioral phase change to landscape structure, demonstrating how resource distribution and vegetation patterns influence aggregation dynamics and gregarization thresholds (Bouaïchi et al. 1996; Collett et al. 1998; Despland and Simpson  2000a, 2000b, 2005a, 2005b, 2006; Despland et al. 2000; Despland 2001, 2003; Despland et al. 2004). Additional work has explored predator–prey dynamics, demonstrating that gregarious coloration functions as aposematic warning coloration when locusts sequester toxins from host plants (Sword et al. 2000), as well as nutritional regulation of phase-related traits (Simpson et al. 2002). Pathogen interactions and transgenerational immune effects have also been examined (Wilson et al. 2002; Elliot et al. 2003, 2005). The UK has extensive international collaboration networks, particularly with Australia, the USA, France, Germany, and Belgium.

**China:** China ranks second in first-authorship (61 authors, 15.2%) and first in total authorship (436 instances, 25.8%), with a total-to-first-author ratio of 7.1—the highest among all major research nations. This profile reflects a highly collaborative, large-team, and programmatically organized research system, in which coordinated efforts drive large-scale, mechanistic investigation rather than individual or small-group leadership. Research from China has established a comprehensive molecular and systems-level dissection of locust phase polyphenism. Early work laid the foundation through the development of genomic and transcriptomic resources, including EST sequencing (Kang et al. 2004), small RNA profiling (Wei et al. 2009), developmental transcriptomics (Chen et al. 2010), and retroelement characterization (Jiang et al. 2012). The first locust genome was subsequently sequenced and analyzed, revealing expanded gene families and methylation-mediated synaptic plasticity underlying phase change (Wang et al. 2014). Early functional work using these resources identified the catecholamine pathway, particularly dopamine, as critical for behavioral phase transition (Ma et al. 2011).

These resources enabled the identification of regulatory layers underlying phase change, particularly epigenetic mechanisms involving DNA methylation and small RNA networks associated with phase memory (Guo et al. 2011; Yang et al. 2014; Chen, He et al. 2015; Guo et al. 2015; Hou et al. 2015, 2017; Guo, Ma et al. 2018, 2020). Subsequent research has focused on mechanistic dissection of signaling pathways and gene function. Molecular cloning and expression studies identified key regulatory components, including heat shock proteins (Wang et al. 2007), the I element (Guo et al. 2010), and histone-modifying enzyme systems (Guo et al. 2016). Functional studies demonstrated that dopamine signaling plays a central role in phase transition, with distinct dopamine receptors contributing differentially to behavioral change (Guo et al. 2015), and that microRNA-mediated regulation (e.g., miR-133 and miR-9a) modulates aggregation behavior and olfactory attraction (Yang et al. 2014; Guo, Ma et al. 2018). Additional work identified key signaling pathways such as cAMP response element-binding protein B (CREB-B) in locomotor plasticity (Hou et al. 2019) and Dnmt3 in behavioral phase regulation (Hou et al. 2020), as well as the involvement of long noncoding RNAs (Li, Chen et al. 2020).

China’s research has also made major contributions to sensory biology and chemical ecology at the molecular level. The identification of 4-vinylanisole as an aggregation pheromone detected by OR35 provided a direct link between chemical signaling and collective behavior (Guo, Yu et al. 2020), and subsequent studies demonstrated its role in sexual maturation synchrony and conspecific interaction (Chen, Hou et al. 2022; Yang et al. 2023). Additional pheromonal systems, including dibutyl phthalate as a phase-specific sex pheromone (Cui et al. 2024), and functional characterization of odorant-binding proteins and olfactory receptors (Wang et al. 2015; Guo, Ren et al. 2018), further established a detailed molecular architecture of olfactory-driven phase change. Hormonal regulation has also been integrated into this framework, with juvenile hormone shown to suppress aggregation via antennal gene expression (Guo, Song et al. 2020) and octopamine and tyramine identified as regulators of attractive and repulsive behaviors, respectively (Ma et al. 2015).

Beyond signaling pathways, Chinese research has extended into broader physiological domains, including metabolism, development, and immune regulation. Studies identified carnitines as key metabolic regulators of phase transition (Wu et al. 2012) and Syntaxin 1A as a modulator of sexual maturation and egg size (Chen, He et al. 2015), and COP9 signalosome complex subunit 7A (CSN7A) as essential for phase transition through proteomic analysis (Tong et al. 2015). Work on pigmentation revealed a β-carotene-binding protein regulating body-color transition (Yang et al. 2019) and spatial regulation of the activation of transcription factor 2 (ATF2) phosphorylation, contributing to warning coloration (Kang et al. 2023). Additional studies demonstrated phase-dependent immunity (Wang et al. 2013, 2022); microbiological interactions, including the role of *Paranosema locustae* (Shi et al. 2014; Feng et al. 2015; Li, Yin et al. 2020); and parental effects on cold hardiness (Wang et al. 2012). Recent work has increasingly emphasized functional validation and systems integration. Gene-editing approaches have enabled causal testing of candidate genes (Li et al. 2016; Guo, Song et al. 2020; Chen, Tong et al. 2022; Wang et al. 2022; Cui et al. 2024), while translational and posttranscriptional regulation has been implicated through ribosomal protein divergence (Li et al. 2023) and N^6^-methyladenosine (m^6^A)-mediated neural pathways controlling aggregation (Huang et al. 2024). Parental crowding was also shown to synchronize progeny egg hatching via enhanced nuclear export of miR-276 in oocytes (Zhu et al. 2024). Studies have also expanded into species-level and ecological contexts, including density-dependent developmental regulation (Shen et al. 2024) and phase transition in Mongolian locust (*Oedaleus asiaticus)* under high-density stress (Guo et al. 2022). Chinese researchers have international collaborations with partners in the USA, Germany, and Spain.

**Japan:** Japan ranks third in first-authorship (58 authors, 14.5%) and fourth in total authorship (146 instances, 8.6%), with a total-to-first-author ratio of 2.5—the lowest among major research nations. This profile reflects a small-team, investigator-driven research system, characterized by sustained individual research trajectories and relatively limited co-authorship per publication. Japanese research has been particularly influential in establishing the endocrine and developmental basis of phase polyphenism. Early studies demonstrated the effects of photoperiod and density on reproductive traits (Tanaka et al. 1993), and identified the genetic basis of albinism as a single recessive locus involving juvenile hormone and corpora cardiaca factors (Hasegawa and Tanaka 1994). A major breakthrough was the discovery of a dark-color-inducing neuropeptide from the corpora cardiaca (Tanaka and Pener 1994), later chemically identified as [His⁷]-corazonin (Tawfik et al. 1999), providing one of the first phase-specific neuroendocrine regulators in locusts.

Subsequent work systematically characterized the role of corazonin in body-color polymorphism, including its dose- and timing-dependent effects, phylogenetic distribution across insect taxa, and interaction with juvenile hormone (Tanaka 2000a, 2000b, 2000c). This endocrine framework was extended through studies of environmental modulation, including temperature effects (Tanaka 2003), morphometric and sensory regulation via antennal sensilla (Maeno and Tanaka 2004; Maeno et al. 2004), and induction of gregarious traits through corpus cardiacum extracts (Tanaka 2007). Field validation linked these mechanisms to natural outbreaks, demonstrating corazonin-associated phase shifts under ecological conditions (Tanaka et al. 2010). Mid-instar nymph responses to crowding were characterized (Tanaka et al. 2016), while long-term monitoring confirmed the robustness of morphometric phase indicators (Tanaka 2022, 2024).

A defining feature of the Japanese research program is its detailed analysis of maternal and transgenerational effects. Studies demonstrated that parental phase state influences progeny size, number, and coloration (Maeno and Tanaka 2008a, 2008b, 2008c; Tanaka and Maeno 2008), with cumulative transgenerational phase effects (Maeno and Tanaka 2009a). Experimental manipulation revealed mechanisms of maternal regulation, including artificial egg miniaturization (Maeno and Tanaka 2009b), hormonal control via juvenile hormone (Maeno and Tanaka 2009c), and density-dependent developmental pathways such as extra molting (Maeno and Tanaka 2010a). A sensitive maternal stage mediating epigenetic transmission of phase traits was also identified (Maeno and Tanaka 2010b).

Japanese researchers have further dissected environmental and sensory triggers of phase change with high experimental precision. Work demonstrated that phase-specific responses can be induced by tactile stimuli (Maeno et al. 2011), contact chemicals and light (Maeno and Tanaka 2012), and visual stimuli alone (Tanaka and Nishide 2012). Substrate color was identified as a primary determinant of body-color polyphenism (Tanaka et al. 2012), highlighting the importance of environmental context in developmental regulation. Additional studies examined phase-dependent feeding behavior (Maeno and Tanaka 2011), density-dependent mating systems (Maeno et al. 2021), and resource allocation strategies favoring egg size over clutch size (Maeno et al. 2022), linking developmental plasticity to life-history evolution.

Field-based and ecological work has complemented laboratory findings, particularly through long-term studies in Africa. These include demonstrations of crowding-induced egg size manipulation under natural conditions (Maeno et al. 2013, 2020), as well as analyses of embryonic development as a function of egg size (Maeno et al. 2023). Additional contributions include studies of overwintering biology (Yamagishi and Tanaka 2009), regional gregarization dynamics (Shimizu et al. 2012), genetically determined wing dimorphism (Nishide and Tanaka 2013), and species-specific egg hatching strategies (Nishide et al. 2015; Nishide and Tanaka 2016). At the molecular level, Japanese research has validated and extended endocrine mechanisms using modern techniques. RNA interference confirmed the role of corazonin in black pattern formation (Sugahara et al. 2015), while subsequent studies showed constitutive expression of the corazonin gene (Sugahara et al. 2016), identified pathway defects in albino mutants (Sugahara et al. 2017), and characterized downstream transcriptional regulators (Sugahara et al. 2018). The interaction between corazonin signaling and pigmentation pathways was further refined through analysis of yellow protein expression (Sugahara and Tanaka 2018). Japanese researchers have extensive international collaborations with Belgium, France, Israel, and African countries, including Mauritania, Mali, Kenya, and Morocco.

**Belgium:** Belgium ranks fourth in first-authorship (30 authors, 7.5%) and third in total authorship (209 instances, 12.3%), with a total-to-first-author ratio of 7.0—comparable to China. This profile reflects a highly collaborative, large-team research system, but one that is distinct in its role as a functional and translational intermediary between molecular discovery and organism-level biology. Belgian research has been central to the systematic characterization of the locust neuroendocrine system, particularly through large-scale peptidomic and neurochemical mapping. Early work produced one of the first comprehensive catalogs of the locust neuropeptidome, revealing phase-dependent differences in peptide expression and establishing a molecular inventory of the insect brain (Baggerman et al. 2001; Clynen et al. 2002, 2006). This effort included the annotation of novel neuropeptide precursors and the identification of phase-specific peptide profiles (Clynen et al. 2006), providing a foundational resource for subsequent functional studies. Building on this descriptive framework, Belgian researchers identified and characterized multiple neuropeptide systems associated with phase change, as well as the first brain-specific phase-related genes (Rahman, Vandingenen et al. 2003). These include neuroparsins (Claeys et al. 2005, 2006; Badisco et al. 2008; Badisco, Huybrechts et al. 2011; Badisco, Ott et al. 2011), pacifastin-related precursors (Breugelmans et al. 2008), and additional phase-specific peptides, including a 6-kDa phase-related peptide (Rahman et al. 2002, 2008; Rahman, Baggerman et al. 2003; Rahman, Vandingenen et al. 2003) and the characterization of yellow protein regulation by a peptidergic brain factor (Sas et al. 2007). This body of work established a detailed molecular landscape of neuroendocrine signaling underlying phase polyphenism.

A defining transition in the Belgian research program is the shift from descriptive molecular cataloguing to functional and mechanistic analysis. This is exemplified by studies that cloned and deorphanized biogenic amine receptors, demonstrating phase-dependent receptor expression and linking neurochemical signaling directly to behavioral outcomes (Verlinden et al. 2010). Parallel work investigated the physiological role of [His⁷]-corazonin in phase polyphenism, integrating molecular and endocrine perspectives (Schoofs et al. 2000; Hoste, Simpson, Tanaka, De Loof et al. 2002; Hoste, Simpson, Tanaka, Zhu et al. 2002; Hoste et al. 2003, 2006). Belgian researchers have also contributed to the integration of epigenetic and genomic approaches. Evidence was provided that epigenetic mechanisms may precede or interact with endocrine regulation in determining phase state (Boerjan et al. 2011), suggesting a hierarchical relationship between regulatory systems. Subsequent work combined transcriptomic and genomic analyses to further characterize phase-dependent gene expression (Badisco, Huybrechts et al. 2011; Badisco, Ott et al. 2011), culminating in the presentation of the first draft genome assembly of the desert locust (Verlinden et al. 2020). Additional studies demonstrated that expression of the foraging gene is modulated by both developmental stage and nutritional conditions (Tobback et al. 2013), linking gene regulation to ecological context. Recent work has extended this integrative approach to sexual selection, demonstrating that yellow protein-mediated coloration in gregarious males functions as an intrasexual antiharassment signal, working in concert with phenylacetonitrile (PAN) to prevent mistaken male–male mounting during high-density scramble mating (Cullen et al. 2022). Belgium has international collaborations, particularly with the UK, Japan, Germany, South Africa, and the USA.

**Kenya:** Kenya ranks fifth in first authorship (27 authors, 6.8%) and fifth in total authorship (97 instances, 5.7%), with a total-to-first-author ratio of 3.6. This near parity reflects a balanced research system, in which Kenyan scientists both lead and actively participate in collaborative work, rather than occupying purely central or peripheral roles. Much of Kenya’s contribution is anchored in the International Centre of Insect Physiology and Ecology (ICIPE), which has developed a strong research program focused on chemical ecology, behavior, and applied pest management. Foundational studies identified aggregation pheromones and semiochemical mechanisms underlying phase-related behavior (Mahamat et al. 1993, 2000; Obeng-Ofori et al. 1993, 1994; Torto et al. 1994, 1996, 1999; Deng et al. 1996; Njagi et al. 1996; Rai et al. 1997; Niassy et al. 1999; Ely et al. 2006; Rono et al. 2008). These studies established a biochemical and behavioral framework for understanding locust aggregation in ecologically realistic contexts. A key contribution of Kenyan research is the identification and functional characterization of pheromonal systems linked to reproduction and phase change. They identified the maturation-accelerating pheromone and associated physiological indicators (Mahamat et al. 1993, 2000; Mahamat, Hassanali, and Ferenz 1997; Munyinyi 1997). Complementary work demonstrated that solitarious females emit a volatile sex pheromone that attracts males over distance, indicating that chemical communication mediates mate finding even in the low-density phase (Inayatullah et al. 1994). Rapid shifts in PAN production in response to density changes were demonstrated (Deng et al. 1996; Deng 2002), and hemolymph absorbance ratios were established as sensitive indicators of phase state, particularly in nymphs.

Kenyan researchers have also contributed significantly to understanding maternal and transgenerational signaling mechanisms. The gregarizing signal responsible for maternal transfer of phase traits was identified as C-8 unsaturated ketones (Malual et al. 2001), providing direct evidence of chemical mediation of phase inheritance. Additional work examined mate location mechanisms and phase-specific mating preferences (Ely et al. 2006), as well as biochemical differentiation between phases, including phase-specific differences in lipid mobilization and adipokinetic hormone sensitivity (Ogoyi et al. 1996), fat body lipid metabolism (Ogoyi et al. 1998) and embryo-level biochemical responses to pheromonal cues (Khamis et al. 2015). Importantly, Kenyan research is characterized by a strong applied orientation, linking fundamental discoveries to pest management strategies. Studies demonstrated phase-independent responses to aggregation pheromones in adults (Njagi et al. 1996), and contributed to the development of semiochemical-based control approaches. Kenya has international partnerships with South Africa, the Czech Republic, the USA, and Ethiopia.

**Israel:** Israel ranks sixth in first-authorship (24 authors, 6.0%) and seventh in total authorship (81 instances, 4.8%), with a total-to-first-author ratio of 3.4. This profile resembles that of the UK in structure, reflecting a moderately collaborative, leadership-oriented system with strong emphasis on mechanistic and experimental approaches. Israeli research has been foundational in establishing the neuroendocrine regulation of phase transition and reproduction. Early work elucidated the role of juvenile hormone and endocrine signaling in phase-related physiology (Pener 1967; Pener and Lazarovici 1979), laying the groundwork for later studies integrating hormonal, behavioral, and neural perspectives. Collaboration with Japanese researchers led to key advances in understanding [His⁷]-corazonin (Tanaka and Pener 1994), highlighting Israel’s role in cross-national endocrine research.

Subsequent work expanded into behavioral neuroscience and neurophysiology, examining the neural basis of gregarious behavior and collective motion (Ayali and Pener 1995; Ayali, Golenser et al. 1996; Ayali, Pener, and Girardie 1996; Ayali, Pener, Sowa et al. 1996; Fuchs et al. 2003; Ayali et al. 2004; Geva et al. 2010; Guershon and Ayali 2012; Golov et al. 2018). Notably, short-term crowding experiments demonstrated that brief exposure (30 minutes) can induce long-lasting behavioral changes dependent on protein synthesis, suggesting that phase transition involves mechanisms analogous to learning and memory (Geva et al. 2010). Israeli researchers have also contributed to chemical communication and reproductive biology, particularly through studies of cuticular hydrocarbons and pheromone-mediated signaling (Heifetz et al. 1994, 1996, 1997, 1998; Applebaum et al. 1997). Structural biology approaches further examined neuropeptide conformation and function (Shalev et al. 2003), linking molecular structure to physiological activity. More recent work has expanded into microbiome research, demonstrating that phase transition is associated with shifts in gut and integument microbial communities (Lavy et al. 2019, 2020, 2022). These studies showed that solitary locusts acquire specific bacterial taxa upon crowding, suggesting a role for microbial dynamics in phase plasticity. Israel has international collaborations with Germany, the UK, and the USA.

**Germany:** Germany ranks seventh in first-authorship (17 authors, 4.2%) and sixth in total authorship (88 instances, 5.2%), with a total-to-first-author ratio of 5.2, indicating a moderately collaborative and institutionally structured research system. German research is distinguished by its strong integration of chemical ecology, sensory neurobiology, and computational modeling. Early work established foundational insights into endocrine regulation and physiological differentiation, including the roles of juvenile hormone and ecdysone (Schneider and Dorn 1994; Schneider et al. 1995; Wiesel et al. 1996), as well as phase-specific hemolymph protein expression (Wedekind-Hirschberger et al. 1999). This was followed by major advances in chemical ecology, particularly the identification of benzyl cyanide and PAN as key pheromonal signals involved in courtship inhibition and social regulation (Seidelmann et al. 2000, 2003, 2005; Seidelmann and Ferenz 2002; Stahr and Seidelmann 2016).

Recent high-impact studies have significantly advanced the mechanistic understanding of olfactory signaling and social behavior. Experimental work combining CRISPR-Cas9 gene editing, behavioral assays, and neurophysiology demonstrated that gregarious locusts produce PAN as a density-dependent anticannibalistic pheromone, detected via the olfactory receptor LmOR70a, which suppresses conspecific cannibalism (Chang, Cassau et al. 2023). Complementary research showed that locust olfactory systems exhibit low redundancy, with narrowly tuned odorant receptors that encode phase-, sex-, and stage-specific behavioral valence, and CRISPR-Cas9 knockout of a specific receptor confirmed its causal role in mediating attraction and aversion (Chang, Unni et al. 2023).

Further advances have elucidated the neurophysiological basis of pheromone detection, demonstrating that the sensory neuron membrane protein SNMP1 is essential for sensitive detection of PAN, with knockout mutants showing reduced electrophysiological responses, altered antennal lobe activity, and impaired behavioral avoidance (Lehmann et al. 2024). At the systems level, recent work revealed synergistic olfactory processing in gregarious locusts, where combined food and social odor cues enhance neural responses and behavioral decisions, a mechanism absent in solitarious individuals (Petelski et al. 2024). German research has also made major contributions to the mechanistic and computational analysis of collective behavior. Recent experimental and virtual reality studies demonstrate that coordinated swarm alignment depends primarily on sensory information quality rather than density, with visual input identified as both necessary and sufficient for coordinated movement (Sayin et al. 2025). These findings support a cognitive vectorial representation model of swarm behavior, challenging classical density-driven interpretations. Additional work has contributed to genomic and epigenetic characterization of locust phase biology (Falckenhayn et al. 2013), as well as to the understanding of visual neurobiology, particularly polarization vision and skylight-based navigation (el Jundi and Homberg 2012). Germany has international collaborations with China, the USA, Belgium, Israel, and the UK.

**Australia:** Australia ranks eighth in first-authorship (16 authors, 4.0%) and ninth in total authorship (63 instances, 3.7%), with a total-to-first-author ratio of 3.9, indicating a balanced research structure with both leadership and collaboration. Australian research has been strongly shaped by field ecology and species-specific investigations. Early work examined morphometric variation in locust phase polymorphism (Blackith 1957). Later studies demonstrated that migration, rather than density dependence, drives population dynamics in Australian plague locust, *Chortoicetes terminifera* (Farrow 1982), while the spur-throated locust, *Austracris guttulosa*, lacks classical phase polymorphism (Elder 1996). A key contribution has been the integration of field ecology with experimental behavioral analysis.

Research established that *C. terminifera* exhibits density-dependent phase polyphenism driven primarily by tactile stimulation (Gray et al. 2009; Cullen et al. 2010). Studies of collective motion revealed structural properties of marching bands and the influence of food distribution on aggregation thresholds (Buhl et al. 2011; Georgiou et al. 2021). Australian researchers have also contributed to epigenetics and gene regulation, demonstrating widespread DNA methylation in locust genomes (Robinson et al. 2011) and differential expression of methylation-related genes between phases (Robinson et al. 2016). Additional work examined stress responses (Chapuis, Simpson et al. 2011), physiological resilience, and methodological standardization for gene expression studies (Chapuis, Tohidi-Esfahani et al. 2011). Australia has international collaborations with the UK, the USA, Canada, Germany, Belgium, and France.

**France:** France ranks 9th in first-authorship (14 authors, 3.5%) and 10th in total authorship (62 instances, 3.7%), with a total-to-first-author ratio of 4.4, indicating a balanced and collaborative research profile. French research has been central to understanding population dynamics, transgenerational effects, and field ecology of phase change. Early studies demonstrated that parental density determines offspring developmental type (Albrecht and Blackith 1957) and that maternal crowding produces cumulative, extra-chromosomal effects across generations (Albrecht et al. 1959). Subsequent work investigated physiological and biochemical mechanisms, including juvenile hormone dynamics (Fuzeau-Braesch et al. 1982), octopamine signaling (Redouane and Fuzeau-Braesch 1985), and cuticular hydrocarbon composition differences between phases (Genin et al. 1986, 1987). Cohesion pheromones promoting aggregation were identified (Fuzeau-Braesch et al. 1988).

A defining feature of French research is its long-term integration with African field systems, particularly in Madagascar and the Sahel. These studies established natural occurrence of gregarious populations, density thresholds for phase change, and genetic variation in gregarization propensity (Franc et al. 2005; Chapuis et al. 2008). Fieldwork on the red locust (*Nomadacris septemfasciata*) in Madagascar further characterized density-dependent color polyphenism and estimated a field gregarization threshold of approximately 10 hoppers per square meter (Lecoq et al. 2011). Further studies have also shown that parental crowding causes transgenerational effects, with daughters reproducing earlier and producing larger offspring (Chapuis et al. 2010). Field-integrated work in Mauritania further demonstrated that gregarious mothers produce fewer but larger eggs with higher lipid reserves, and their hatchlings exhibit enhanced starvation tolerance—a maternal bet-hedging strategy (Maeno et al. 2013). Quantitative analyses of spatial distribution patterns (Cisse, Ghaout, Mazih, Jourdan‐Pineau et al. 2015) and field models linked vegetation structure and density thresholds to phase transition (Cisse, Ghaout, Mazih, Ould Babah Ebbe et al. 2015). France has international collaborations with Japan, the UK, Ukraine, Australia, the USA, Mali, Mauritania, and Morocco.

**United States of America (USA):** The USA ranks 10th in first-authorship (12 authors, 3.0%) and 8th in total authorship (64 instances, 3.8%), with a total-to-first-author ratio of 5.3, indicating a moderately collaborative system. The USA research has been particularly influential in developing evolutionary, comparative, and theoretical frameworks for locust phase polyphenism. Early comparative work demonstrated that the non-swarming American bird grasshopper (*Schistocerca americana*) exhibits reduced and geographically variable density-dependent behavioral plasticity compared to the classic locust, the desert locust (*Schistocerca gregaria*) (Sword 2003). Phylogenetic studies established evolutionary relationships and transitions underlying phase traits (Song 2005; Song and Wenzel 2008; Song et al. 2017). Subsequent comparative work across species demonstrated variation in the dynamics of phase change and clarified the role of corazonin in morphological but not behavioral traits (Foquet and Song 2021; Foquet et al. 2021, 2022). Additional studies characterized phenotypic plasticity in *Schistocerca cancellate* (Pocco et al. 2019). The USA researchers have also contributed to nutritional ecology and behavioral theory, demonstrating that nutrient imbalance influences swarm formation (Cease et al. 2010, 2017, 2023), and cannibalism can drive the evolution of behavioral phase polyphenism (Guttal et al. 2015). Mathematical models have further defined density thresholds and hysteresis effects in gregarization (Topaz et al. 2012). The USA has international collaborations with Germany, the UK, Australia, China, Argentina, and Mexico.

#### Patterns of international authors’ collaborations

**Fig. 4:**
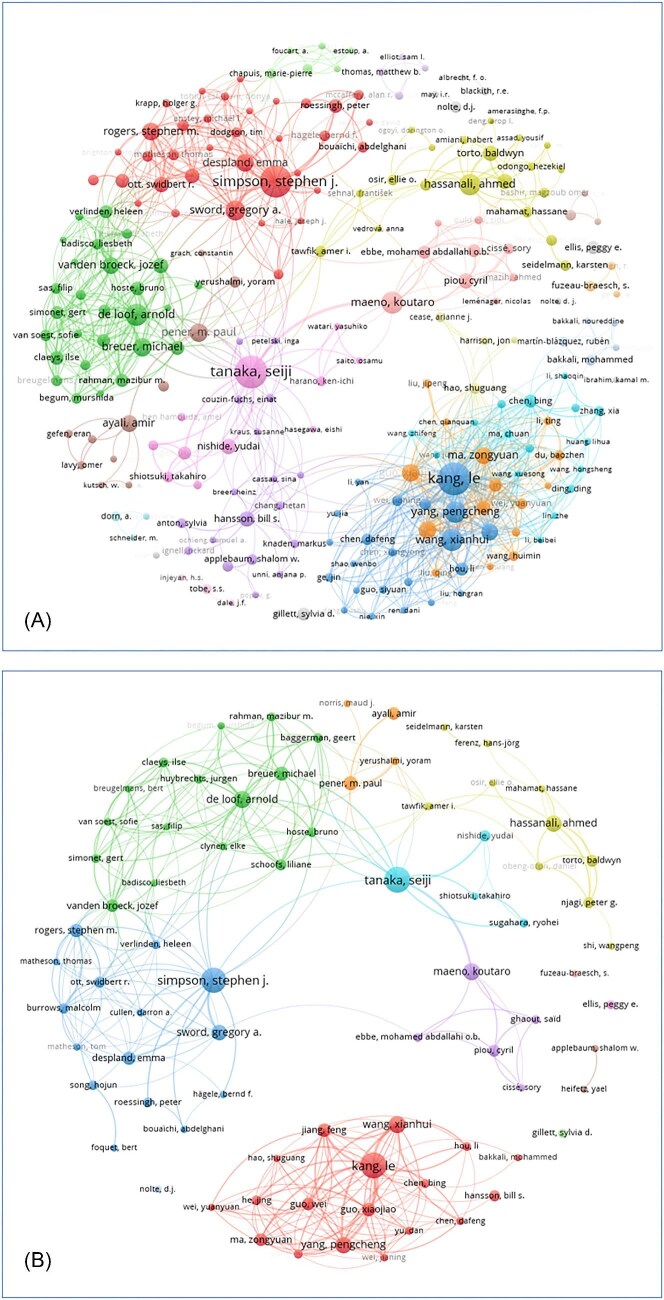

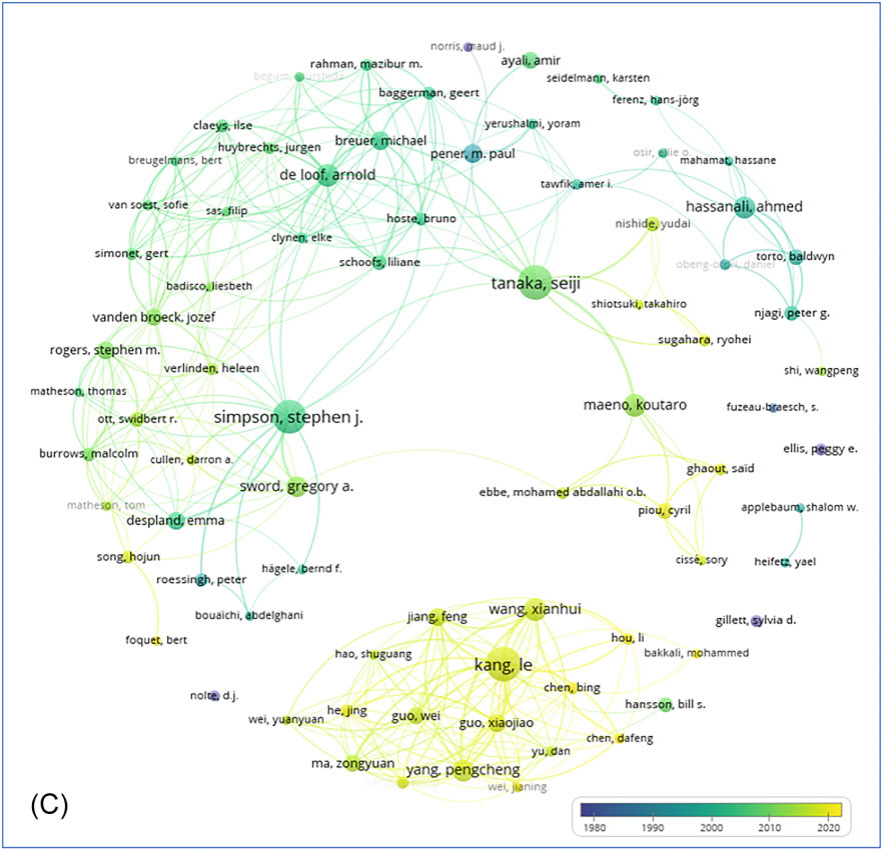
Co-authorship network visualizations of locust phase polyphenism research. (**A**) Network analysis of authors with two or more publications. (**B**) Network highlighting authors with five or more publications. Node size is proportional to publication count; edge thickness indicates co-authorship strength; colors differentiate clusters of frequent collaborators. (**C**) Temporal overlay indicating publication year (darker = older studies, lighter = newer).

The co-authorship network reveals a hub-and-spoke architecture characterized by pronounced structural asymmetries across national research systems. Large, densely connected domestic clusters are evident in the UK, China, Japan, Belgium, Kenya, and Israel (see Fig. 4). These clusters correspond to high-degree nodes and reflect research systems with strong internal co-authorship density and sustained institutional continuity. Although cross-hub collaboration is clearly present, it remains less dense than intra-cluster connectivity, indicating partial geographical and disciplinary compartmentalization among major research centers. This pattern suggests that, despite increasing international collaboration, knowledge production retains a degree of structural segmentation, with core research programs developing largely within nationally or institutionally bounded networks. Several countries—including Germany, France, Australia, and the USA—play critical brokerage roles within the global network. These systems exhibit relatively high betweenness centrality, functioning as bridges that connect otherwise weakly linked clusters and facilitate cross-regional knowledge exchange. Their role is defined less by internal dominance than by connective capacity, enabling the circulation of ideas across distinct research traditions.

Within the Chinese network, the presence of multiple color-coded substructures indicates a high degree of internal modularity rather than fragmentation. These modules represent differentiated collaboration groups operating within a centralized national system, linked through shared authorship around key contributors. This configuration reflects a cohesive yet internally specialized research architecture, in which multiple subprograms are coordinated through overlapping collaborative ties. Despite China’s extensive international engagement, the network remains visually and structurally domestically centered, reflecting the predominance of internal co-authorship relative to cross-national links. More broadly, the network reveals a persistent core–periphery structure in global collaboration. With the notable exception of the Kenyan cluster—centered on the ICIPE and strengthened by sustained institutional investment since the 1990s—most researchers from locust-affected regions, including countries across the Sahel, the Horn of Africa, and parts of the Middle East, remain weakly integrated. These actors typically appear as peripheral nodes with low degree and betweenness centrality. This pattern reflects a two-tiered system of scientific integration: a limited number of research centers achieve deep, continuous participation in global knowledge production, while most range-state researchers are incorporated only intermittently, often as secondary collaborators rather than leaders of independent research programs. Figure 4 illustrates this structure, highlighting a globally connected yet stratified and functionally differentiated network, in which dense national hubs, internally modular substructures, and bridging connections collectively shape the architecture of collaboration in locust phase polyphenism research.

### Keyword co-occurrence, thematic trends, and research gaps

The keyword co-occurrence network revealed a central cluster of terms focused on phase change mechanisms, with phase polyphenism (occurrence = 85), phase change (occurrence = 62), phase polymorphism (occurrence = 62), phenotypic plasticity (occurrence = 50), and phase transition (occurrence = 33) as the most frequent concepts. These terms were predominantly linked to two model species: *S. gregaria* (occurrence = 149), *L. migratoria* (occurrence = 88), along with their common names, desert locust (occurrence = 43) and migratory locust (occurrence = 15). The network showed progression from behavioral terms—behavior (occurrence = 25), aggregation (occurrence = 24), behavioral plasticity (occurrence = 13), and gregarization (occurrence = 34)—toward physiological and molecular terms. Specific regulatory factors appeared with notable frequencies: juvenile hormone (occurrence = 23), serotonin (occurrence = 18), corazonin (occurrence = 13), neuropeptide (occurrence = 13), his^7^-corazonin (occurrence = 11), adipokinetic hormone (occurrence = 8), and ecdysteroid (occurrence = 6). Chemical ecology terms were also present: pheromone (occurrence = 18), aggregation pheromone (occurrence = 16), PAN (occurrence = 13), guaiacol (occurrence = 6), and phenol (occurrence = 6). Behavioral phase-state terms included gregarious (occurrence = 22), solitarious (occurrence = 14), solitary (occurrence = 8), aggregation behavior (occurrence = 5), social aggregation (occurrence = 6), and olfaction (occurrence = 10). The combined frequency of solitarious and solitary was 44, compared to 22 for gregarious alone.

**Fig. 5:**
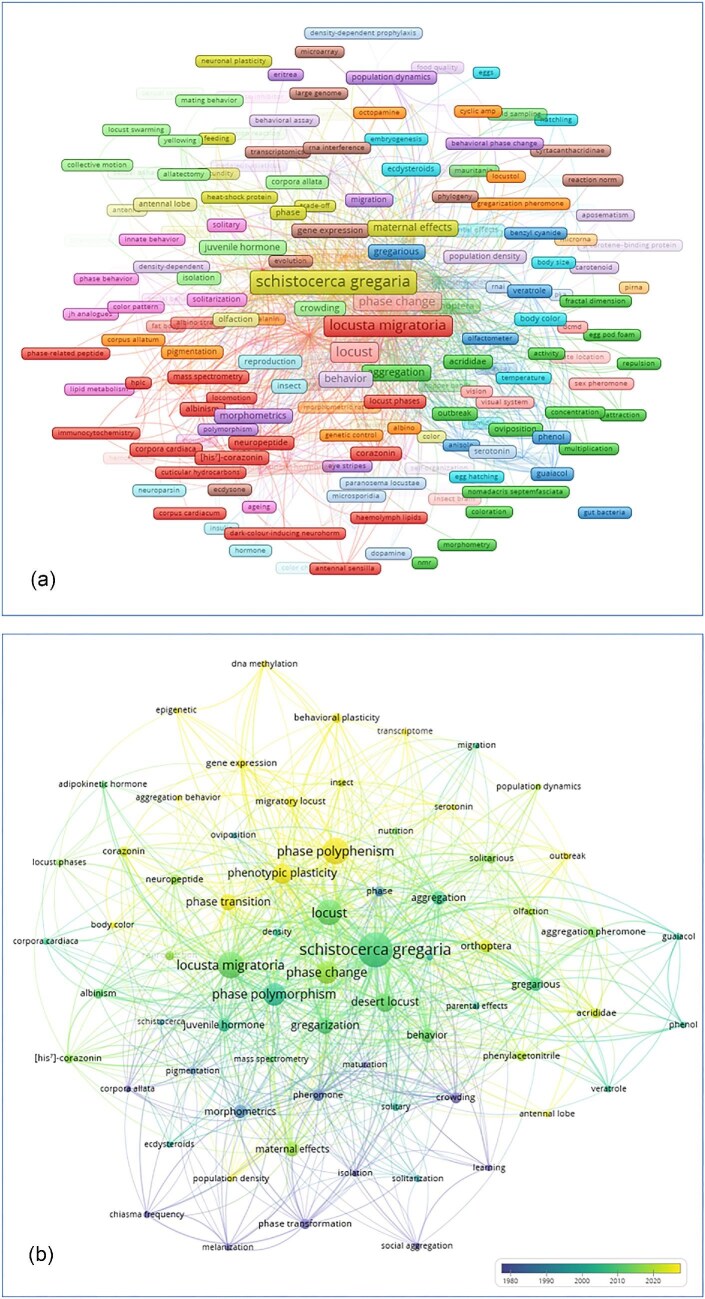
**(A)** Co-occurrence analysis of all keywords. Node size reflects frequency; edge thickness indicates co-occurrence strength; colors represent clusters of related terms. **(B)** Co-occurrence network of keywords appearing more than five times with a temporal overlay indicating publication year (darker = older, lighter = newer). Node size reflects frequency; edge thickness indicates co-occurrence strength.

Methodological terms included gene expression (occurrence = 12), transcriptome (occurrence = 5), RNA interference (occurrence = 4), CRISPR/Cas9 (occurrence = 4), epigenetics (occurrence = 5) and DNA methylation (occurrence = 7), maternal effects (occurrence = 27), and maternal inheritance (occurrence = 3). Classical phenotypic terms appeared as well: morphometrics (occurrence = 21), body color (occurrence = 5). Ecological and population-level terms showed lower frequencies: reproduction (occurrence = 12), population density (occurrence = 7), outbreak (occurrence = 7), and population dynamics (occurrence = 6). Environmental variables included temperature (occurrence = 4) and humidity (occurrence = 3). Terms related to climate change, microbiome, or gut bacteria were absent or had very low frequencies, for example, microbiome and gut bacteria (occurrence = 2). Species other than the two dominant models were rarely represented: *C. terminifera* (occurrence = 3), *N. septemfasciata* (occurrence = 3), *O. asiaticus* (occurrence = 2), and *Schistocerca piceifrons* (occurrence = 1). Temporal overlay of keywords (Fig. 5) indicated that older publications concentrated on physiological and behavioral themes, while newer publications introduced transcriptomic and genetic manipulation terms.

### Leading journals in locust phase polyphenism research

**Fig. 6:**
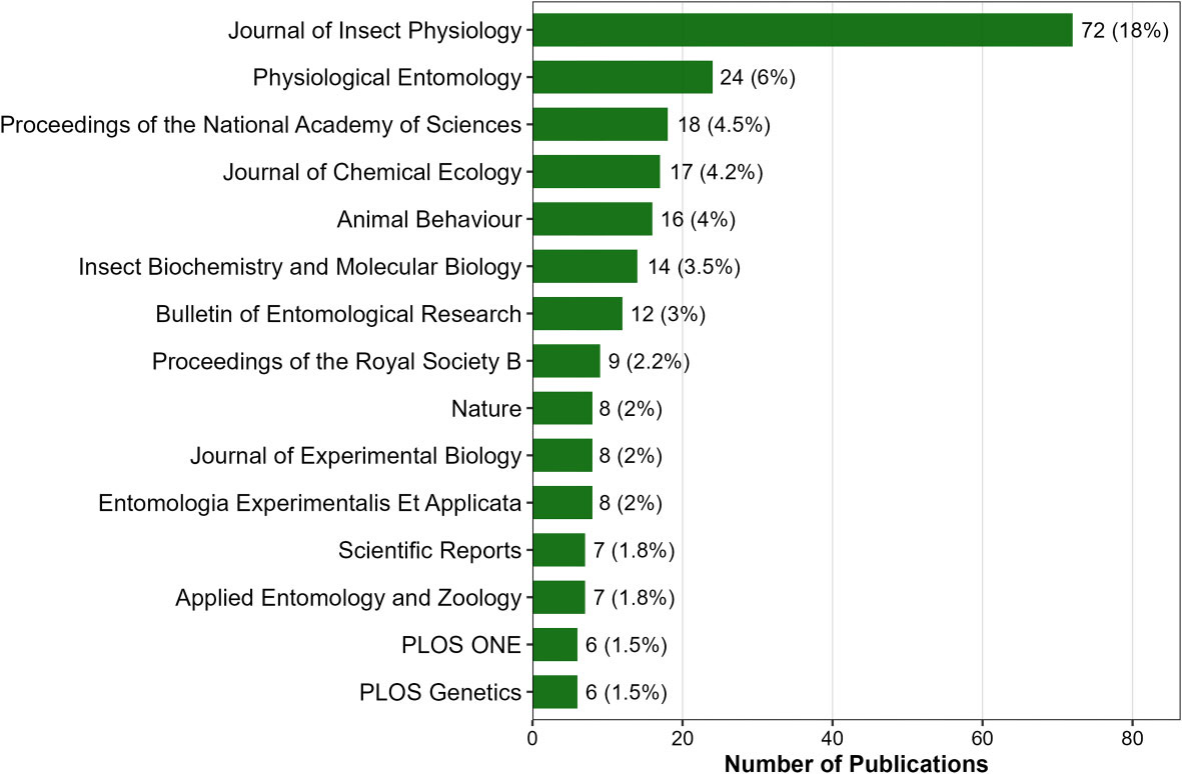
Top 15 leading journals in locust phase polyphenism research.

The analysis of the top publishing journals revealed the distribution shown in Fig. 6. *Journal of Insect Physiology* published the most papers (*n* = 72, 18.0% of the corpus). *Physiological Entomology* ranked second (*n* = 24, 6.0%). High-impact multidisciplinary and general biology journals included *Proceedings of the National Academy of Sciences* (*n* = 18, 4.5%), *Proceedings of the Royal Society B* (*n* = 9, 2.2%), and *Nature* (*n* = 8, 2.0%). Specialized journals included *Journal of Chemical Ecology* (*n* = 17, 4.2%), *Animal Behaviour* (*n* = 16, 4.0%), *Insect Biochemistry and Molecular Biology* (*n* = 14, 3.5%), *Bulletin of Entomological Research* (*n* = 12, 3.0%), *Journal of Experimental Biology* (*n* = 8, 2.0%), *Entomologia Experimentalis et Applicata* (*n* = 8, 2.0%), *Applied Entomology and Zoology* (*n* = 7, 1.8%), and *PLOS Genetics* (*n* = 6, 1.5%). Broad-scope journals were represented by *Scientific Reports* (*n* = 7, 1.8%) and *PLOS One* (*n* = 6, 1.5%). These data confirm that locust research maintains deep specialization in physiology, behavior, and chemical ecology journals while achieving regular visibility in high-impact multidisciplinary venues.

## Discussion

### General interpretation of the results

This bibliometric analysis reveals a field characterized by substantial mechanistic detail alongside persistent structural constraints that shape both knowledge production and its applicability. Three interrelated patterns emerge from the data: a narrow taxonomic focus, pronounced geographical asymmetry, and a disconnect between mechanistic understanding and real-world outbreak dynamics. These patterns define the current limits of locust phase polyphenism research and indicate key directions for its advancement. A central finding is the overwhelming concentration of research on two model species—*S. gregaria* and *L. migratoria*. These species account for the majority of species-specific keyword occurrences in the dataset, while other locust taxa appear only sporadically. Although this focus is justified by their economic importance and experimental tractability, it has produced what may be termed a model species paradox: the field has achieved detailed mechanistic understanding within a narrow subset of taxa yet remains uncertain whether these mechanisms generalize across the broader diversity of locusts. Comparative transcriptomic evidence reinforces this limitation, demonstrating that fewer than 10% of phase-related differentially expressed genes are shared between the two dominant species (Bakkali et al. 2024). This suggests that much of the current knowledge base reflects species-specific pathways rather than general principles, limiting predictive generalization across ecological contexts and evolutionary lineages. This taxonomic concentration is closely intertwined with a second structural feature: the emergence of distinct national research orientations shaped by outbreak history and institutional mandates. Countries experiencing recurrent locust outbreaks tend to prioritize applied, management-oriented research, whereas nations without direct outbreak pressure predominantly pursue fundamental, curiosity-driven science.

A third defining feature is the pronounced geographical asymmetry in research leadership. The co-authorship network exhibits a core–periphery structure in which a small number of countries dominate first authorship and agenda-setting, while most researchers from locust-affected regions remain peripherally integrated. This pattern is consistent with network metrics showing dense, high-degree clusters in a limited number of countries and low centrality for most range-state researchers. Kenya represents a notable exception, demonstrating that sustained institutional investment, capacity building, and equitable partnership design can enable range-state researchers to achieve meaningful leadership in locust research. This asymmetry extends beyond issues of equity and has direct implications for scientific validity. Locust population dynamics are governed by highly localized ecological factors—including soil conditions, vegetation structure, microclimate, and land-use practices (Cisse et al. 2013; Cease et al. 2015; Meynard et al. 2020). Such factors are most deeply understood by range-state researchers, who work within outbreak regions. When such locally embedded knowledge is underrepresented, models of locust dynamics are constructed on incomplete ecological foundations, limiting their predictive accuracy and applicability to real-world outbreak conditions. This pattern indicates the need for research systems that enable range-state scientists to define and lead research agendas, rather than participating primarily as secondary collaborators.

The keyword analysis identifies several thematic blind spots that reinforce the disconnect between mechanistic knowledge and ecological reality. Most striking is the complete absence of “climate change” from the keyword network, despite the role of climatic variables in shaping locust outbreaks. The absence of an integrated climate framework indicates that detailed physiological knowledge remains insufficiently connected to predictive models of outbreak dynamics under changing environmental conditions. A similar imbalance is reflected in the low frequency of “outbreak” as a keyword. This suggests that the field has focused extensively on how phase change occurs while giving comparatively limited attention to when and why outbreaks emerge in natural systems. Mechanistic insights derived from controlled experimental settings are not consistently linked to ecological processes operating at landscape and regional scales.

Additional blind spots emerge in biological and transgenerational domains. Despite early evidence that gut bacteria contribute to aggregation pheromone production (Dillon et al. 2002), microbiome research remains relatively sparse, though recent work has begun to explore mechanistic links between crowding and microbiome shifts (Lavy et al. 2022). Similarly, while maternal effects (occurrence = 27) are well established (Islam et al. 1994a, 1994b; Bouaïchi et al. 1995; McCaffery et al. 1998; Chapuis et al. 2008, 2010; Maeno and Tanaka 2008c; Wang et al. 2012; Chen, Li et al. 2015), the mechanistic basis of maternal inheritance remains unresolved. It remains unclear whether maternal programming requires prolonged crowding or occurs rapidly, and whether transmission is mediated by egg foam chemistry or epigenetic mechanisms (Islam et al. 1994a, 1994b; McCaffery et al. 1998; Robinson et al. 2016; Nishide and Tanaka 2019). These patterns indicate that key phenomena are well documented, but their causal mechanisms remain insufficiently understood.

At the same time, the field is undergoing a methodological transition from descriptive profiling to causal and functional analysis. Transcriptomic studies have revealed the scale of phase-related plasticity, generating high-resolution hypotheses about underlying mechanisms (Bakkali and Martín-Blázquez 2018). However, establishing causality requires experimental validation. Functional genomic tools such as RNA interference and CRISPR/Cas9 are beginning to enable direct manipulation of candidate genes and signaling pathways (Li et al. 2016; Chang, Unni et al. 2023), although their representation in the keyword network remains low, indicating early-stage adoption. Epigenetics represents a particularly promising frontier for explaining phase memory and transgenerational persistence of traits. Mechanisms such as DNA methylation and histone modification provide plausible pathways for these effects, although systematic experimental validation remains limited. The microbiome similarly represents an underexplored domain where manipulative approaches could clarify whether microbial communities act as drivers or correlates of phase change. These structural, thematic, and methodological imbalances point toward several strategic priorities for reorienting the field.

## Recommendations

Based on the patterns and gaps identified, we propose five strategic priorities to reorient locust-phase biology research. First, we recommend establishing comparative research consortia—coordinated, multi-laboratory initiatives funded to examine phase transition mechanisms in understudied species. These consortia would employ standardized assays and omics protocols to enable direct cross-species comparisons, testing whether mechanisms identified in model species are conserved, divergent, or convergent. Second, we recommend integrating phase biology with climate science through a dedicated research program that systematically examines how climate variables interact with known phase mechanisms. This would involve controlled environment experiments, spatial modeling linking climate projections to gregarization probability, and integration of climate data with long-term outbreak records to identify changing outbreak regimes.

Third, we strongly recommend prioritizing functional validation over further descriptive omics expansion. While transcriptomic and epigenomic profiling have generated valuable hypotheses, systematic investment is now required in approaches such as CRISPR-mediated knockout of epigenetic regulators, microbiome manipulation experiments, pharmacological intervention studies, and longitudinal studies tracking phase memory across generations. Fourth, we emphasize sustained investment in range-state institutional capacity, building on successful models such as ICIPE. Development agencies and research funders should support the establishment or strengthening of locust research centers in under-represented outbreak regions through sustained funding for salaries, infrastructure, and PhD training, coupled with equitable partnership agreements that ensure co-determination of research agendas.

Fifth, we emphasize the need for integrative, cross-scale research frameworks that explicitly link molecular mechanisms to ecological dynamics and applied management. Recent initiatives such as the Behavioral Plasticity Research Institute (BPRI)—a USA National Science Foundation–funded virtual institute coordinating research from molecules to landscapes across multiple Schistocerca species—represent promising structural models for achieving this integration. Importantly, this model addresses several structural limitations identified in this study, including fragmentation across scales, over-reliance on single-species systems, and weak integration between fundamental and applied research. Scaling such frameworks to include a broader diversity of locust species and stronger participation from range-state researchers will be critical for aligning mechanistic insight with real-world outbreak dynamics.

### Limitations

This study has several limitations that qualify its conclusions and suggest directions for future bibliometric work. The restriction to English-language publications introduces a systematic bias that likely underrepresents research from key range-state regions. Future bibliometric analyses should address this by incorporating regional databases and non-English sources or employing multilingual search strategies. Additionally, the exclusion of grey literature, and management-oriented publications likely obscures policy-relevant research and operational knowledge, including documentation of outbreak dynamics, control efficacy, and local ecological knowledge produced by locust control programs in range-state nations that never enter indexed journals. Database coverage limitations further compound these gaps, as reliance on PubMed, Web of Science, and Scopus—while comprehensive for English-language science—may exclude specialized entomological databases and regional indexes that could enrich future analyses.

Moreover, despite efforts to design comprehensive search strings, they may not capture all relevant studies due to variations in terminology, evolving keywords, or discipline-specific jargon; future research would benefit from closer collaboration with senior researchers and experts in locust phase polyphenism to refine search strategies and explore the literature more deeply. A further important scope limitation concerns the focus of our search strategy. Our Boolean terms were deliberately designed to capture research on phase polyphenism specifically—its mechanisms, correlates, and drivers—rather than the broader literature on locust control, monitoring, operational management, or outbreak forecasting. Consequently, our finding of a “disconnect” between mechanistic research and applied challenges (e.g., the absence of climate change keywords) should be interpreted as a pattern within the phase polyphenism literature itself, not as a claim about the entire universe of locust-related research. It is likely that FAO technical reports, national control program documents, and applied entomology journals contain substantial work on climate impacts, outbreak prediction, and management outcomes that our search strategy was not designed to retrieve. The disconnect we document, therefore, lies between the fundamental science of phase polyphenism and the practical challenges of outbreak management, rather than indicating an absence of applied research elsewhere.

## Conclusion

This bibliometric analysis reveals a field whose substantial achievements are structurally constrained. The past three decades have produced a deep mechanistic understanding of phase change in two model species, with discoveries published in the most selective journals. However, co-authorship networks expose significant geographical disparities, with research leadership concentrated in a small number of countries, while most other locust-affected regions remain peripherally integrated. Keyword mapping identifies critical blind spots—climate change, socioeconomic dimensions, non-model species, and microbiome causality—reflecting a disconnect between mechanistic knowledge and the complex challenges of outbreak dynamics in a changing world. Quantitative network metrics reveal persistent structural asymmetries even as international collaboration increases. The structural constraints documented here—narrow species focus, geographic inequity, and thematic blind spots—reflect modifiable features of how research is organized and supported. Addressing them will require strategic reorientation: from reliance on a few model species to comparative ecology, from episodic collaboration to equitable partnerships with range-state scientists, and from descriptive approaches to functional validation across diverse taxa, including systematic investigation of underexplored areas such as epigenetic regulation and microbiome interactions. This bibliometric mapping provides an evidence-based framework to guide these efforts.

## Supplementary Material

obag018_Supplemental_File

## Data Availability

The search strings are provided as supplementary file. The bibliometric dataset is available from the corresponding author upon reasonable request.
