# Supplementary material for: The Integrative Imperatives of Locust Phase Polyphenism Research: A Bibliometric Analysis
Source: Integr Org Biol. 2026 May 6;8(1):obag018. doi: 10.1093/iob/obag018 (PMC13168895; doi:10.1093/iob/obag018)
Supplement: obag018_Supplemental_File [file obag018_supplemental_file.docx]

**Lists of search strings used across the database**

**Web of Sciences**

("locust" OR "Schistocerca" OR "Locusta migratoria" OR "Nomadacris" OR "Chortoicetes"OR "Oedaleus" OR "Austracris") AND ("phase polyphenism" OR "phase polymorphism" OR "phase change" OR "phase transition" OR "phenotypic plasticity" OR "density-dependent" OR "gregari*" OR "solitar*" OR "phase characteristic*") AND ("behavi*" OR "morphometr*" OR "colo*" OR "pheromone" OR "juvenile hormone" OR "corazonin" OR "serotonin" OR "transcriptom*" OR "microbiome")

**Scopus**

TITLE-ABS-KEY ( "Locust" OR " Schistocerca" OR "Locusta migratoria" OR "Nomadacris" OR "Chortoicetes" OR "Oedaleus" OR “Austracris” ) AND TITLE-ABS-KEY ( "phase polyphenism" OR "Phenotypic plasticity" OR " phase polymorphism" OR "density-dependent" OR "phase change" OR "gregari*" OR "solitar*" ) AND TITLE-ABS-KEY ( "behavio*" OR "morphometr*" OR "juvenile hormone" OR "corazonin" OR "transcriptom*" OR "microbiome") AND ( LIMIT-TO ( SUBJAREA , "AGRI" ) OR LIMIT-TO ( SUBJAREA , "NEUR" ) OR LIMIT-TO ( SUBJAREA , "BIOC" ) OR LIMIT-TO ( SUBJAREA , "IMMU" ) ) AND ( LIMIT-TO ( DOCTYPE , "ar" ) ) AND ( LIMIT-TO ( LANGUAGE , "English" ) )

**PubMed**

(Locust* OR Schistocerca OR "Locusta migratoria" OR Nomadacris OR Chortoicetes OR Oedaleus OR Austracris) AND (phase polyphenism OR "phenotypic plasticity" OR density-dependent OR gregarious OR solitary OR solitarisation) AND (behavior* OR morphometr* OR "juvenile hormone" OR corazonin OR transcriptom* OR microbiome).
